# Randomized trial of dentists’ understanding: treatment benefit in absolute numbers vs relative risk reduction

**DOI:** 10.1590/1807-3107bor-2024.vol38.0070

**Published:** 2024-08-05

**Authors:** Paulo NADANOVSKY, Branca Heloisa de OLIVEIRA, Ronaldo LIRA-JUNIOR, Ana Paula Pires dos SANTOS

**Affiliations:** (a) Fundação Oswaldo Cruz – Fiocruz, National School of Public Health, Department of Epidemiology, Rio de Janeiro, RJ, Brazil; (b) Universidade do Estado do Rio de Janerio – UERJ, School of Dentistry, Department of Community and Preventive Dentistry, Rio de Janeiro, RJ, Brazil.; (c) Karolinska Institute, Department of Dental Medicine, Stockholm, Sweden.

**Keywords:** Communication Barriers, Persuasive Communication, Data Interpretation, Statistical

## Abstract

This study aimed to assess whether dentists correctly understand the benefit of a dental treatment when it is presented using absolute numbers or relative risk reduction (RRR). This parallel-group randomized controlled trial recruited dentists from 3 postgraduate courses in Rio de Janeiro, Brazil. Participants received, in sequentially numbered sealed opaque envelopes, the description of a hypothetical scenario of the benefit (avoidance of multiple tooth loss) of nonsurgical periodontal treatment without or with antibiotics. Treatment benefit was presented in 2 different formats: absolute numbers or RRR. Dentists were given 10 minutes to read the treatment scenario and answer 5 questions. The final sample for analysis included 101 dentists. When asked to estimate the number of patients out of 100 who would avoid multiple tooth loss *without* antibiotics, 17 dentists (33%) in the absolute numbers group and 12 (25%) in the RRR group provided the correct response (p = 0.39). Regarding treatment with antibiotics, 26 dentists (50%) in the absolute numbers group and 14 (29%) in the RRR group provided the correct response (p = 0.04). Only 16 dentists (31%) in the absolute numbers group and 12 (25%) in the RRR group gave correct answers for both questions (p = 0.51). Most dentists did not correctly understand the benefit of the treatment, irrespective of the format it was presented. Slightly more dentists correctly understood the benefit of the treatment when it was presented as absolute numbers than as RRR.

## Introduction

Several challenges hinder the translation of clinical trial results into meaningful benefits for patients. The challenges include not only the use of surrogate outcomes and the presence of reporting biases but also the reliance on relative measures to convey the effects of a treatment. These issues can impede the accurate assessment of treatment benefit and limit its practical impact on patient outcomes.^1^ Additionally, the way statistical information is presented, whether related to the efficacy of a treatment, the risk of disease occurrence, or the accuracy of a diagnostic test, can lead readers to different conclusions and ultimately result in poor decisions in clinical practice.^2^

When interpreting the results of diagnostic or screening tests, it is easier for health professionals, including dentists, to understand the probability of having a disease after a positive test (the positive predictive value) using absolute numbers.^3,4^ Absolute numbers, sometimes referred to as natural frequencies,^5^ provide the actual number of people in a population who have the disease and the number of people who tested positive, rather than relying on conditional probabilities expressed as single percentages.

Similarly, the benefit of a treatment can be communicated in multiple ways, including relative and absolute effect measures. However, medical findings often emphasize relative measures in media reports,^6,7^ health materials,^8^ and journals,^9-13^ which can lead to exaggerated depictions of a treatment benefit. A relative risk reduction (RRR) of 50% may be interpreted to mean that the incidence of an outcome was 20% in one group and 10% in the other, or 2% and 1%, 0.2% and 0.1%, and so on. The ongoing debate centers around determining the most effective numerical format for conveying treatment benefits, considering factors such as the recipient’s level of numeracy, the use of visual aids, and the provision of base-rate information.^3,14^

Health professionals should intervene only when there is solid evidence that a treatment improves the natural history of a disease,^15^ which refers to the disease course without any intervention. For example, without treatment, periodontitis may lead to multiple tooth loss. Periodontal treatment may improve the natural history of periodontitis if it reduces the risk of multiple tooth loss. The rate of multiple tooth loss in patients with periodontitis who do not receive periodontal treatment may be referred to as the base rate (or baseline rate). To assess whether a treatment effectively improves the natural history of a disease, it is crucial that all health care stakeholders, including dentists, grasp the importance and magnitude of treatment effect estimates. Unfortunately, many health professionals find it challenging to accurately interpret and effectively communicate these estimates.^3^

The objective of this study was to assess whether dentists correctly understand the benefit of a dental treatment when it is presented in the form of either absolute numbers or RRR.

## Methods

### Ethics, registration, and report

The Ethics Committee of the University of the State of Rio de Janeiro approved this study (CAAE60115416.5.0000.5259), and each participant provided informed consent by signing a consent form. We registered the study protocol at ClinicalTrials.gov (NCT03051295) and adhered to the CONSORT guidelines in reporting our findings.^16^ Additionally, this trial was conducted alongside a similar study that examined dentists’ inferences about diagnostic accuracy data.^4^

### Study design

Parallel-group randomized controlled trial.

### Settings

Rio de Janeiro, Brazil, from July to December 2016.

### Participants

Dentists recruited from 3 postgraduate courses.

### Randomization

Blocks of 6 using a computer-generated table of random numbers with an equal allocation ratio.

### Interventions

All participants received written instructions outlining the study’s objectives. They were then tasked with completing a questionnaire to gather details such as their date of birth, sex, years since graduation, specialty degree, field of expertise, and workplace. Subsequently, each participant was given 2 consecutively numbered sealed opaque envelopes that contained the description of 2 hypothetical scenarios: one focused on the accuracy of bite-wing radiographs (previously published elsewhere^4^) and the other related to the benefit of periodontal treatment, both without and with antibiotics. The scenario pertaining to periodontal treatment included a set of 5 accompanying questions. Treatment benefit (avoidance of multiple tooth loss) was presented in 2 distinct formats: absolute numbers or RRR. Both groups received information about the base rate, which is the risk of multiple tooth loss in patients with periodontitis who do not receive treatment, representing the natural history of periodontitis. The base-rate information was presented as absolute numbers for the absolute numbers group and as percentages for the RRR group.

The first and second questions tasked participants with rating the effectiveness of the treatment without and with antibiotics on a Likert scale, spanning from 1 (not effective at all) to 10 (extremely effective). The third question sought the participant’s opinion on recommending the addition of antibiotics to nonsurgical periodontal treatment, with response options ranging from 1 (would not recommend) to 10 (would strongly recommend). The fourth and fifth questions were open-ended and prompted participants to estimate the number of patients with periodontitis who would potentially benefit from treatment (avoid multiple tooth loss) without or with antibiotics. A comprehensive description of the clinical scenario and the associated questions answered by the dentists is provided in the Box.

A time limit of 10 minutes was imposed for reading the treatment scenario, answering the 5 questions about treatment benefit, and returning the envelope.

### Outcomes

The primary outcome was dentists’ understanding, which was determined by their accuracy in answering questions 4 and 5 related to the hypothetical scenario. Specifically, they were asked about the number of patients with periodontitis (out of 100 in our hypothetical scenario) who would avoid multiple tooth loss due to nonsurgical periodontal treatment, both without and with antibiotics. The correct answers were 1 and 2, respectively.

Secondary outcomes included dentists’ perception of treatment benefit, which was assessed through their subjective ratings (questions 1 and 2). Additionally, the study evaluated persuasiveness, measured by the dentists’ willingness to recommend the addition of antibiotics to nonsurgical periodontal treatment (question 3).

### Blinding

The outcome assessors and those responsible for administering the questionnaire were kept unaware of each participant’s group assignment. However, due to the nature of the study, participants themselves were aware of their assigned group and therefore could not be blinded. Nonetheless, they remained unaware of the specific hypothesis being tested and the specific comparisons under investigation. This approach ensured that all parties involved were effectively blinded, including outcome assessors, investigators, and participants.

### Sample size

Based on an expected proportion of correct answers of 12% and 35% in the RRR and absolute numbers groups, respectively ^7^, we calculated that we would need 51 participants per group to detect a significant difference between the groups, with α = 0.05 and power = 0.80.

### Statistical analysis

The data were recorded and organized in a Microsoft Excel spreadsheet. We used SPSS 17.0 for statistical analysis and performed t-tests and Fisher exact tests. The level of significance was set at 5%.

## Results

**Figure 1:**
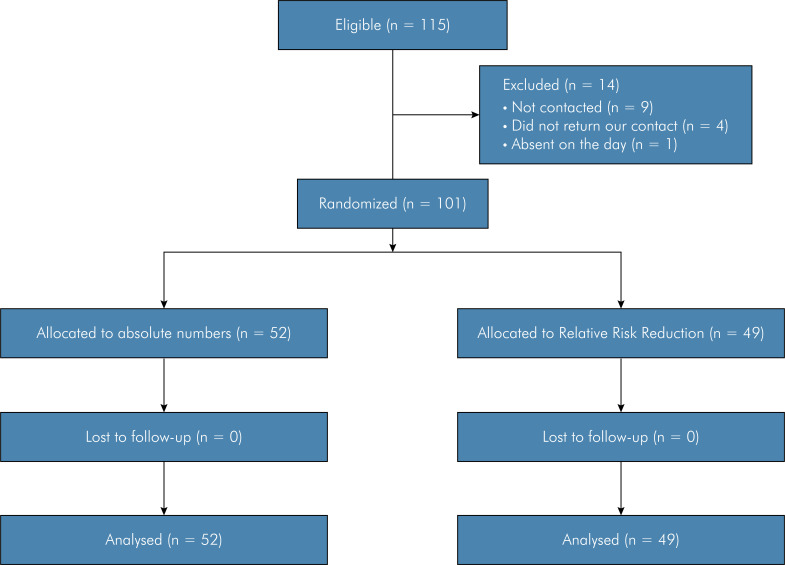
Flow diagram showing the number of dentists who were eligible, excluded with reasons, randomized, and analyzed.

Out of the initial pool of 115 eligible participants, 9 could not be reached due to logistical challenges, 4 did not respond to our attempts to contact them, and 1 was absent during questionnaire administration. As a result, the final sample for analysis included 101 dentists. Figure 1 illustrates the flow diagram of the trial, presenting the number of dentists who were eligible, excluded (with reasons), randomized, and ultimately analyzed in this study.

Out of the total participants, 75 were women (74.3%). The mean age of the participants was 29 years (SD 5.9). On average, they had 5.3 years of professional experience (SD 5.1). Most participants worked exclusively in private practice (53; 52.5%) and held a specialty degree (82; 81.2%). Dentists were from different specialties, including orthodontics, periodontics, endodontics, stomatology, prosthodontics, restorative dentistry, pediatric dentistry, and oral and maxillofacial surgery. Table 1 presents the baseline characteristics of the participants in the absolute numbers and RRR groups.

**Table 1:** Baseline characteristics of the dentists in the groups receiving information about periodontal treatment benefit (hypothetical scenario) using absolute numbers or relative risk reduction.

Variable	Absolute numbers	Relative risk reduction

	n = 52	n = 49
Age – mean (SD)	28.8 (5.8)	29.2 (6.1)
Years since graduation – mean (SD)	5.1 (4.6)	5.5 (5.6)
Women – n (%)	41 (78.8)	34 (69.4)
Specialty degree – n (%)	42 (80.8)	40 (81.6)
Working exclusively in clinical practice – n (%)	25 (48.1)	28 (57.1)

SD = standard deviation.

When asked to estimate the number of patients out of 100 who would avoid multiple tooth loss due to nonsurgical periodontal treatment *without* antibiotics (correct answer was 1), 17 dentists (32.7%) in the absolute numbers group and 12 (24.5%) in the RRR group provided the correct response (p=0.39). Regarding treatment with antibiotics (correct answer was 2), 26 dentists (50.0%) in the absolute numbers group and 14 (28.6%) in the RRR group provided the correct response (p = 0.04). Only 16 dentists (30.8%) in the absolute numbers group and 12 (24.5%) in the RRR group provided correct answers for both questions (p = 0.51) (Table 2). In the RRR group, 34 dentists (69.4%) significantly overestimated the treatment benefit compared with 23 dentists (45.1%) in the absolute numbers group. By overestimation we mean that they believed that 20 or more patients would avoid multiple tooth loss, instead of the correct estimates of 1 and 2, without and with antibiotics, respectively.

**Table 2:** Frequencies of correct answers among the dentists in the groups receiving the information about periodontal treatment benefit (hypothetical scenario) using absolute numbers and relative risk reduction.

Variable	Absolute numbers	Relative risk reduction	p-value ^c^

	n = 52	n = 49	

	n (%)	n (%)	
Correct answer question 4 ^a^	17 (32.7)	12 (24.5)	0.39
Correct answer question 5 ^b^	26 (50.0)	14 (28.6)	0.04
Correct answer questions 4 and 5	16 (30.1)	12 (24.5)	0.51

a: Question 4: “For each 100 adult patients with periodontitis, how many will avoid multiple tooth losses due to the non-surgical periodontal treatment *without* antibiotics?” Correct answer = 1; b: Question 5: “For each 100 adult patients with periodontitis, how many will avoid multiple tooth losses due to the non-surgical periodontal treatment associated *with* taking antibiotics?” Correct answer = 2; c: Pearson Chi-Square, exact significance two-sided test.

**Figure 2:**
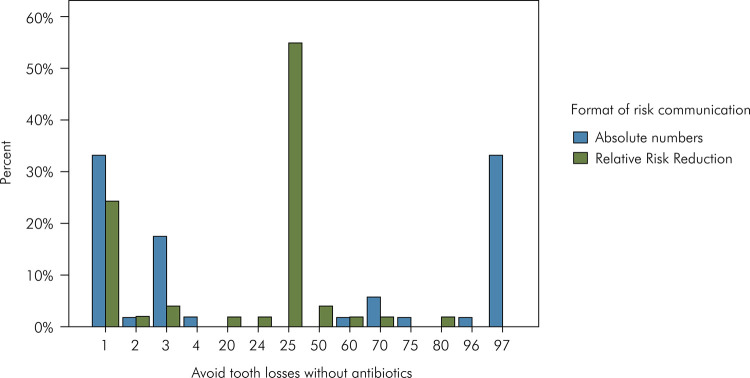
Frequency of answers to the question “For each 100 adult patients with periodontitis, how many will avoid multiple tooth losses due to the non-surgical periodontal treatment *without* antibiotics?”. Base-rate was four with multiple tooth losses, and correct answer was one will avoid it.

**Figure 3:**
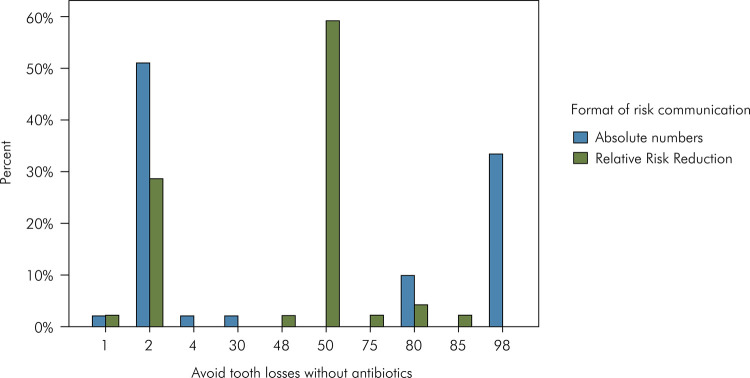
Frequency of answers to the question “For each 100 adult patients with periodontitis, how many will avoid multiple tooth losses due to the non-surgical periodontal treatment associated *with* taking antibiotics?”. Base-rate was four with multiple tooth losses, and correct answer was two will avoid it.

In the scenario without antibiotics, where the correct answer was 1, the most frequent answers in the absolute numbers group were 1 and 97, whereas in the RRR group the most frequent answer was 25 (Figure 2). Regarding the scenario with antibiotics, where the correct answer was 2, the most common answers in the absolute numbers group were 2 and 98, whereas in the RRR group the most frequent answer was 50 (Figure 3).

The average subjective rating of perceived treatment benefit given by dentists in the absolute numbers group was higher for both treatment without antibiotics (6.1, SD 2.1) and treatment with antibiotics (7.0, SD 2.0) than the average rating given by dentists in the RRR group (4.5, SD 1.9 and 6.3, SD 1.6, respectively). The mean differences were 1.6 (SE 0.4) and 0.7 (SE 0.4), respectively (Table 3). No statistically significant differences were found between the groups in terms of whether the participants would recommend adding antibiotics to nonsurgical periodontal treatment (persuasiveness). The ratings were 6.1 (SD 3.0) in the absolute numbers group and 7.0 (SD 2.9) in the RRR group, with a mean difference of -0.8 (SE 0.6) (Table 3).

**Table 3:** Perception of effectiveness (1 to 10) and persuasiveness regarding the prescription of antibiotics (1 to 10) in periodontal treatment (in a hypothetical scenario), among dentists in the groups receiving the information about treatment effectiveness using absolute numbers and relative risk reduction.

Variables	Absolute numbers	Relative risk reduction	p- value ^d^

	n = 52	n = 49	

	Mean (SD)	Mean (SD)	
Perception of benefit without antibiotics ^a^	6.1 (2.1)	4.5 (1.9)	0.01
Perception of benefit with antibiotics ^b^	7.0 (2.0)	6.3 (1.6)	0.05
Persuaded to prescribe antibiotics ^c^	6.1 (3.0)	7.0 (2.9)	0.15

SD = standard deviation. a: In this hypothetical scenario, how would you rate the effectiveness of non-surgical periodontal treatment *without* antibiotics to prevent multiple tooth loss? Mark one option on the scale below, being 1 no effectiveness and 10 extremely effective?; b: In this hypothetical scenario, how would you rate the effectiveness of non-surgical periodontal treatment *with* antibiotics to prevent multiple tooth loss? Mark one option on the scale below, being 1 no effectiveness and 10 extremely effective?; c: In this hypothetical scenario, would you recommend adding antibiotics to non-surgical periodontal treatment? Mark one option on the scale below, being 1 would not recommend and 10 would strongly recommend; d: t-test for equality of means, significance (2-tailed).

## Discussion

The main finding of this study was that most dentists did not correctly understand the benefit of a dental treatment when it was presented either as absolute numbers or in the form of RRR. Slightly more dentists correctly understood the benefit of the treatment when it was presented as absolute numbers than as RRR.

However, when analyzing incorrect answers provided by the dentists, a valuable insight emerged. The incorrect answers revealed that the dentists’ confusion and misinterpretation of the treatment benefit stemmed from their inability to consider the base rate presented in the hypothetical scenario, that is, the rate of multiple tooth loss in patients with periodontitis who do not receive periodontal treatment. Failure to do so is indicative of base-rate neglect,^4,12^ and it was evident in both the absolute numbers group, where the base rate was expressed as 4 in 100, and the RRR group, where the base rate was expressed as 4%.

In the RRR group, many dentists focused on the information that treatment efficacy was 25% and 50%, assuming that, for every 100 patients, 25 and 50 would avoid multiple tooth loss in the scenarios without and with antibiotics, respectively. However, they failed to grasp that these percentages needed to be applied to the baseline risk of 4% for multiple tooth loss. Therefore, 25% of 4% corresponds to 1 patient out of 100, and 50% of 4% corresponds to 2 patients out of 100.

In a similar vein, many dentists in the absolute numbers group followed a flawed rationale. They based their reasoning on the fact that, out of 100 patients, 3 and 2 would experience multiple tooth loss in the scenarios without and with antibiotics, respectively. From this information, they concluded that the remaining patients, 97 and 98, respectively, would benefit from the treatment by assuming that all 100 patients received the treatment. However, they overlooked the crucial point that most patients, specifically 96 individuals, would not have experienced multiple tooth loss even without any treatment. Thus, these dentists fell into the misconception that every patient who was treated and did not experience the outcome automatically benefited from the treatment.

This misconception was slightly more pronounced in the RRR group than in the absolute numbers group. Additionally, a significantly larger number of dentists in the RRR group greatly overestimated the treatment benefit. It appears that, when presented with absolute numbers, fewer dentists neglected the base-rate information. It has been suggested that, whenever RRR is communicated, the base rate should also be provided to prevent misleading interpretations.^3^ Previous research has shown that including the base rate significantly improves the understanding of absolute risk reduction (ARR), but it does not have the same impact on RRR.^17^

The optimal numerical format to enhance understanding of treatment benefits remains uncertain. Previous studies have yielded conflicting results, indicating that the effectiveness of different formats may vary depending on factors such as the specific population under study, the participants’ level of numeracy, the inclusion of the base rate in the information provided, and the utilization of visual aids or illustrations.^3,14,17,18^

Among the general public, percentages were found to be the most effective format for conveying absolute differences in treatment benefits ^14^. However, the study conducted by Woloshin and Schwartz in 2011 did not specifically evaluate the RRR format. In their study, participants received treatment benefit estimates in percentages, along with the base rate and incidence in the control and test groups. For instance, they presented information such as “3.3% of individuals had a heart attack in the control group compared to 2.5% in the test group,” compared with providing natural frequencies such as “33 in 1000” and “25 in 1000” in the control and test groups, respectively. However, the study did not examine whether the conventional RRR format was more effective than natural frequencies in communicating treatment effects, as no participant was provided with the RRR of 24%, that is, [1-(2.5/3.3)]. Therefore, the question of whether the RRR format is superior or inferior to absolute numbers in conveying treatment benefits remained unaddressed.

In our study, the RRR group was presented with the base rate of the disease (i.e., 4% of patients will experience multiple tooth loss). However, unlike the study conducted by Woloshin and Schwartz in 2011,^14^ we provided the RRR values (25% and 50%, without and with antibiotics, respectively). This allowed us to directly compare the RRR format with the absolute numbers format. Although we did not include an RRR group without the base rate of the disease, it could be a valuable consideration for future research, as this is a format commonly used by epidemiologists and pharmaceutical companies.

The existing research on risk communication indicates that understanding remains low regardless of the numerical format used. To enhance understanding and reasoning, the inclusion of visual aids may be promising, such as bar charts and icon arrays.^18,19^ However, to effectively utilize these visual aids, it is crucial to have a thorough understanding of the numbers that will be used to create them. This knowledge serves as a foundation for effective communication strategies.

Evidence syntheses have shown that relative effect estimates, such as RRR, may lead people to perceive treatment as more beneficial, compared with ARR. Health professionals and lay people were also more persuaded to prescribe or undergo a treatment, respectively, when presented with results expressed as RRR than as ARR ^3 18^. Unexpectedly, in our study dentists in the absolute numbers group perceived the benefit of the treatment as being higher than those in the RRR group (though we used absolute numbers, not the ARR format). However, it is unclear whether the mean differences observed between the groups are meaningful, as they correspond to 1.6 and 0.7 points on a 1-10 Likert scale for the perceived benefit of the treatment without and with antibiotics, respectively. Our study also assessed persuasiveness, defined in this context as willingness to recommend antibiotics. Notably, there was no discernible difference in persuasiveness between the groups.

The findings of this study highlight a crucial issue that requires attention when assessing dentists’ understanding of treatment benefit. It is essential for dentists to recognize that, even when providing effective treatment, usually only a small percentage of patients will benefit from it, as many patients may naturally recover or remain stable without intervention. The concerning aspect of our findings is that a significant number of dentists mistakenly believed that a large proportion of patients, such as 25, 50, 97, or 98 out of 100, would benefit from the treatment, whereas in the provided scenario, only 1 or 2 patients would benefit from it. This misconception raises serious concerns, as these misguided beliefs have the potential to contribute to widespread overdiagnosis and overtreatment in the field of dentistry.

Limitations of this study include the following: the comparison tested in this study was conducted as part of a larger randomized controlled trial that examined not only dentists’ understanding of treatment benefit but also their diagnostic reasoning. The sample size calculation was primarily based on the diagnostic reasoning aspect, which may have impacted the statistical power for the treatment benefit assessment. Additionally, choices made in hypothetical scenarios may not always align with decisions made in real-life situations, introducing a potential discrepancy. Furthermore, the lack of a comparison group receiving the RRR format without the base rate of the disease, commonly used by epidemiologists and pharmaceutical companies, limits the comprehensiveness of the study findings.

To improve communication and understanding, future research should explore additional approaches, such as emphasizing that patients often recover or remain stable even without treatment, thereby highlighting the modest probability of treatment benefit. The use of visual aids and qualitative techniques, such as focus groups, could be valuable in this regard. Furthermore, future comparisons should include at least 3 distinct groups: RRR without the base rate, absolute numbers with the base rate, and percentages with the base rate. This comprehensive approach will provide a more robust assessment of effective communication strategies for treatment benefit information.

## Conclusion

Most dentists did not correctly understand the benefit of a dental treatment (avoidance of multiple tooth loss) when it was presented either as absolute numbers or in the form of RRR. Slightly more dentists correctly understood the benefit of the treatment when it was presented as absolute numbers than as RRR. It appears that dentists in both groups have overlooked the base rate of multiple tooth loss in our hypothetical scenario.
